# Prolonged activation of EP3 receptor-expressing preoptic neurons underlies torpor responses

**DOI:** 10.21203/rs.3.rs-2861253/v1

**Published:** 2023-05-02

**Authors:** Natalia L. S. Machado, Francesca Raffin, Satvinder Kaur, Alexander S. Banks, Nicole Lynch, Oleksandra Fanari, Oscar R. Plascencia, Sydney Aten, Janayna D. Lima, Sathyajit S. Bandaru, Richard D. Palmiter, Elda Arrigoni, Clifford B. Saper

**Affiliations:** 1. Department of Neurology, Beth Israel-Deaconess Medical Center and Harvard Medical School, Blackfan circle, Boston 02215 USA; 2. Division of Endocrinology, Metabolism, and Diabetes, Beth Israel-Deaconess Medical Center and Harvard Medical School, Boston 02215 USA; 3. Howard Hughes Medical Institute and Department of Biochemistry, University of Washington, 1959 NE Pacific St., Seattle WA 98195-7370 USA

## Abstract

Many species use a temporary drop in body temperature and metabolic rate (torpor) as a strategy to survive food scarcity. A similar profound hypothermia is observed with activation of preoptic neurons that express the neuropeptides Pituitary Adenylate-Cyclase-Activating Polypeptide (PACAP)^1^, Brain Derived Neurotrophic Factor (BDNF)^2^, or Pyroglutamylated RFamide Peptide (QRFP)^3^, the vesicular glutamate transporter, Vglut2^4,5^ or the leptin receptor^6^ (LepR), estrogen 1 receptor (Esr1)^7^ or prostaglandin E receptor 3 (EP3R) in mice^8^. However, most of these genetic markers are found on multiple populations of preoptic neurons and only partially overlap with one another. We report here that expression of the EP3R marks a unique population of median preoptic (MnPO) neurons that are required both for lipopolysaccharide (LPS)-induced fever^9^ and for torpor. These MnPO^EP3R^ neurons produce persistent fever responses when inhibited and prolonged hypothermic responses when activated either chemo- or opto-genetically even for brief periods of time. The mechanism for these prolonged responses appears to involve increases in intracellular calcium in individual EP3R-expressing preoptic neurons that persist for many minutes up to hours beyond the termination of a brief stimulus. These properties endow MnPO^EP3R^ neurons with the ability to act as a two-way master switch for thermoregulation.

Drugs that block the formation of prostaglandins, including non-steroidal anti-inflammatories and acetaminophen, are widely used clinically to prevent or reduce fever. Fever produced by immune stimuli such as LPS depends upon the action of prostaglandin E2 on the EP3R expressed by neurons in the median preoptic nucleus^9^. As the EP3R is inhibitory, it has been hypothesized that it must inhibit neurons that tonically act to reduce core body temperature (Tb). Recent studies have found that preoptic neurons that express the EP3R overlap extensively with those producing PACAP and QRFP which have been proposed as markers for neurons that promote hypothermia and produce torpor in mice^1,3,8,10^. However, each of these peptides is produced by a number of different populations of preoptic neurons, and the identity of the core population of neurons responsible for torpor remains unknown. We hypothesized that EP3R marks a core population of hypothermic neurons that work as a thermoregulatory switch responsible for both torpor and inflammatory fever^10^.

## PACAP-expressing MnPO neurons mediate inflammatory fever

**Fig. 1.**
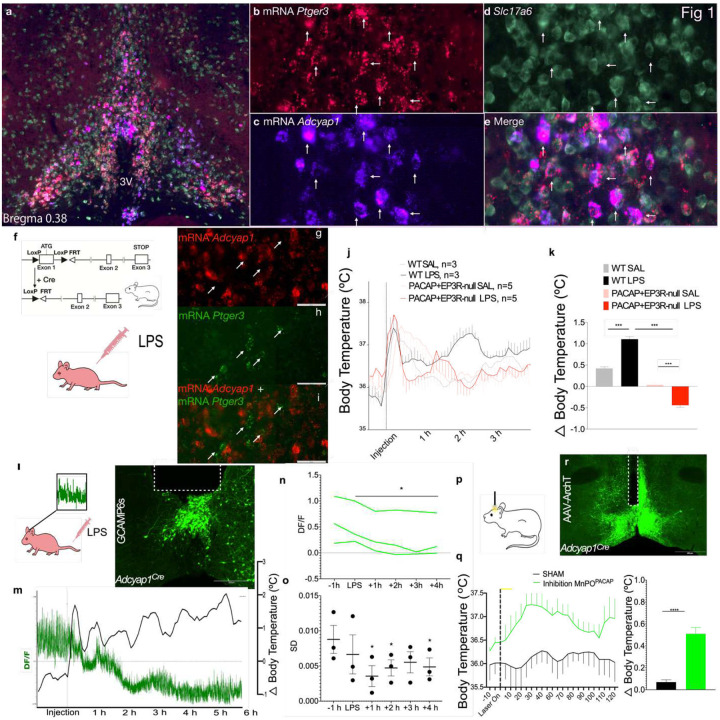
The overlap of EP3R and PACAP expression reveals a unique population of neurons that are critical for mediating fever. RNA-scope in situ hybridization (ISH) showed the overlap of EP3R (red, *Ptger3* gene) and PACAP (blue, *Adcyap1* gene) in the MnPO of a Vglut2-IRES-Cre-L10 mouse (green, *Slc17a6*) (a). The colocalization of *Ptger3, Adcyap1* and *Slc17a6* is clear at a higher magnification (b-e). We found about 45% of the MnPO^PACAP^ neurons co-expressing EP3R and 61% of EP3R neurons expressing PACAP. We crossed *Ptger3*^*flox/flox*^ mice with *Adcyap1*^*Cre*^ mice to cause deletion of EP3R from PACAP+ neurons (PACAP+EP3R-null mice) (f). ISH showed successful deletion of EP3R (green) from MnPO^PACAP^ (red) neurons, and a remaining subset of MnPO^EP3R+/PACAP-^ neurons (white arrows) (g-i). In WT mice, after the first peak of Tb elevation caused by the stress, mice showed a typical fever response 1–3h after LPS injection. However, in PACAP+EP3R-null mice LPS caused a small hypothermic response 1–3 hr after injection (0.4 °C ± 0.03 WT SAL vs. 1.1 °C ± 0.04 WT LPS vs. 0.03 °C ± 0.04 PACAP+EP3R-null mice SAL vs. −0.4 °C ± 0.04 PACAP+EP3R-null mice LPS, F(3,92)=195.1 One-way ANOVA, followed by Bonferroni’s post hoc test, p<0.0001) (j-k). A schematic figure of the protocol for calcium imaging (left panel) and a representative image of GCAMP6s native fluorescence expression and fiber placement in a *Adcyap1*^*Cre*^ male mouse is shown (right panel) (l). A recording of calcium signaling (green) and Tb (black) of a *Adcyap1*^*Cre*^ mouse illustrates a reduction in fluorescence during fever relative to the baseline (DF/F) (m). Data analyses of 1-h bins of the DF/F before and after LPS injection normalized by the baseline condition show a robust reduction in the DF/F of calcium fluorescence in MnPO^PACAP^ neurons during fever (n=3), and standard variance (SD, i.e., spikiness) of the signal peaks before fever is reduced after the LPS injection (n-o). These results suggest that fever may be caused by the inhibition of hypothermic MnPO^PACAP+^ neurons. To test this, we photoinhibited MnPO^PACAP^ neurons (p). Photoinhibiting MnPO^PACAP^ cell bodies for 15 min (10 stimuli of 60 s on followed by 30 s off) caused a hyperthermic effect that lasted for ~ 1 h (n=4) compared to sham stimulation (0.6 ± 0.09°C SEM MnPO STIM vs 0.06 ± 0.04 °C SEM SHAM STIM, paired t-test, p<0.0001) (q). ArchT-GFP+ expression and optical fiber placement in MnPO neurons of a *Adcyap1*^*Cre*^ mouse (r).

We previously showed that there was extensive overlap in the expression of EP3R and PACAP in the MnPO^11^. However, it was not clear to what extent there might be populations expressing one, but not the other marker, and how important that overlap might be for identifying neurons involved in both fever and torpor responses. To quantify the co-expression, we performed two-color fluorescence in situ hybridization (ISH) for *Adcyap1* (the gene for PACAP) and *Ptger3* (the gene encoding EP3R) mRNA, finding that 60.3% ± 8.8 SEM of MnPO neurons that express *Ptger3* also express *Adcyap1* and that 45.6%+ 2.8 SEM of MnPO neurons that express *Adcyap1* also express *Ptger3* (n=3; Fig 1a–e). This overlap is a bit less than the 77.6% co-expression of *Adcyap1* in MnPO^*Ptger3*^ neurons that we found in a previous study in which we used *Adcyap1*-IRES-Cre::L10-GFP reporter mice to identify PACAP neurons^11^. By either measure this overlap is substantial, but it is possible that the residual neurons expressing one or the other gene, rather than the population with co-expression, could be responsible for the bulk of thermoregulatory properties. To determine the extent to which the overlap in expression of these two markers identifies the neurons that participate in LPS fever responses, we crossed *Ptger3*^*flox/flox*^ mice with *Adcyap*^*Cre*^ mice to generate mice with EP3R selectively inactivated in PACAP neurons (PACAP+EP3R- null mice) and compared LPS fever responses to littermates that did not express Cre as controls (Fig 1f). ISH confirmed successful deletion of *Ptger3* mRNA (green) from *Adcyap1*-expressing (red) preoptic neurons (Fig. 1g–i), but revealed a subset of *Ptger3+/Adcyap1-* neurons (small white arrows in Fig 1i and S Fig 1a–b).

The control and PACAP+EP3R-null male mice showed a similar circadian rhythm of baseline Tb at 22 °C during the light and dark phase (Fig S 1c). Both mouse lines also showed a brief (~45 min) hyperthermia (~ 1.0–1.5 °C) due to the stress from handling during the injection of LPS (20 μg/kg, i.p.) or vehicle (Fig. 1j). Control mice also showed a typical fever response induced by of LPS with an elevation of Tb of about 1.5 °C from 1–3 h after the injection^11^. However, the PACAP+EP3R-null mice had a fall in Tb of ~0.5 °C during this same period (Fig. 1j–k). This decrease in Tb was smaller than the 1.0 °C reduction seen in mice with complete deletion of EP3R in the CNS^9^ after systemic LPS or preoptic injection of prostaglandin E2, suggesting that the remaining EP3R+/PACAP-neurons may have partially masked the hypothermia. In addition, our ISH experiments identified other sites in the brain that co-expressed *Adcyap1* and *Ptger3*, including the ventromedial hypothalamic nucleus and raphe pallidus (RPa) as well as a few cells in the parabrachial and dorsomedial hypothalamic (DMH) nuclei. However, it seems unlikely that these sites contribute to LPS fever, as eliminating EP3R expression in the MnPO alone has been shown to be sufficient to prevent LPS- or PGE2-induced hyperthermia^9^. The evidence therefore supports the hypothesis that the co-expression of EP3R and PACAP in the MnPO marks a population of preoptic neurons that play a critical role in LPS fever responses, but that the EP3R+PACAP- MnPO neurons also may make a small contribution to the hyperthermia induced by LPS.

## PACAP-expressing MnPO neurons are inhibited during inflammatory fever

We hypothesized that PGE2 binding to EP3R, which are mainly inhibitory receptors^12^, would inhibit EP3R/PACAP neurons in the MnPO during LPS fever. To test that hypothesis, we used fiber photometry to investigate the activity patterns of MnPO^PACAP^ neurons during a febrile response in *Adcyap1*^*Cre*^ mice injected with an adeno-associated viral vector carrying a Cre-dependent calcium reporter, AAV-DIO-GCaMP6s.

We monitored changes in intracellular calcium in MnPO^PACAP^ neurons during baseline conditions or fever induced by injection of LPS (Fig 1l–o). The GCaMP signal of MnPO^PACAP^ neurons was strongly reduced for >6 h after LPS, an effect that was associated with the prolonged elevation of Tb (Fig 1 m–o). In addition to the reduced activity of MnPO^PACAP^ neurons during fever, we found a reduction in the variance (a reduction in “spiky peaks”) in calcium signaling in MnPO^PACAP^ neurons after the LPS injection (Fig. 1o–p). MnPO^PACAP^ neurons were previously found to have increased numbers of these spiky peaks during naturally occurring torpor episodes, in which they are activated to cause hypothermia^1^. These results indicate that LPS produces an inhibitory response in MnPO^PACAP^ neurons during LPS fever that is the opposite of their response during torpor, and is consistent with their playing a major role in driving LPS fever.

## Inhibiting MnPO^PACAP^ neurons or their synaptic terminals in the dorsomedial hypothalamus (DMH) or raphe pallidus (RPa) mimics fever

**Fig. 2.**
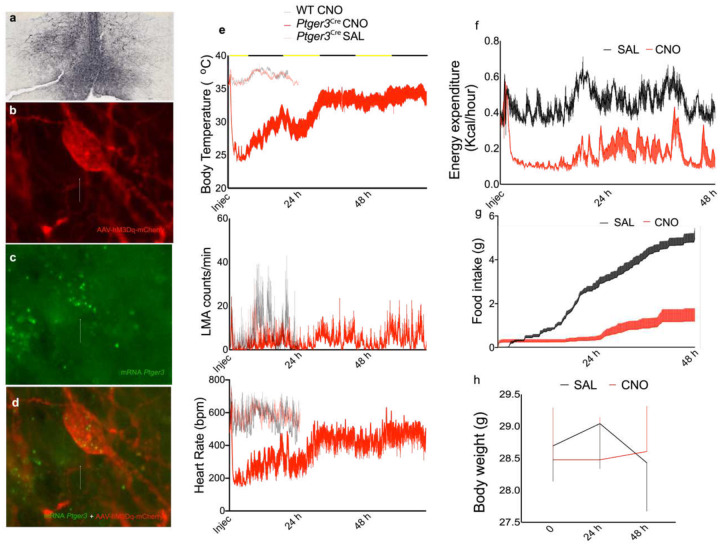
Activation of EP3R-expressing MnPO neurons induces a prolonged but reversible state of deep hypothermia. AAV-DIO-hM3Dq-mCherry (hM3Dq) injection into the MnPO of *Ptger3*^Cre^-cre mice (a) permitted selective activation of the EP3R-expressing MnPO neurons as shown by hM3Dq expression (red, b), only in neurons showing *Ptger3* mRNA (green, c, and merge d). Chemogenetic activation of the MnPO^EP3R^ neurons with CNO 0.3 mg/kg caused hypothermia for up to 60 hrs. The mean Tb during the first 24 h after saline injection (SAL, light red) was 36.6 ± 0.04 °C and after CNO (red) dropped to a mean of 29.0 ± 0.15 °C (paired t-test, p=0.04, n=6, e). This response was associated with a low LMA (4.97 ± 0.22 counts per minute after SAL vs. 2.3 ± 0.14 count per minute after CNO, paired t-test, p<0.0001, n=6) and reduced HR (577 ± 2.6 bpm after SAL vs. 307 ± 5.0 bpm after CNO, paired t-test, p<0.0001, n=6). No hypothermia was found in WT mice treated with CNO (gray) (e). During the first 48 h after 0.3 mg/kg CNO injection, mice show a reduction of their mean Energy Expenditure (0.45 ± 0.003 Kcal/hour after SAL vs. 0.19 ± 0.003 Kcal/hour after CNO, F_(489,2940)_=1.38, two-way ANOVA, followed by Bonferroni’s post hoc test p<0.0001, n=4 each group) (f) and food intake (5.1 ± 0.4 g 48 h after SAL vs. 1.2 ± 0.6 g 48 h after CNO, paired t-test, p=0.002, n=4 each group (g), but not their mean body weight (28.9 ± 0.15 g after SAL vs. 28.7 ± 0.03 g after CNO, paired t-test, p=0.4, n=4 each group, h), confirming a profound hypometabolic state.

To test the hypothesis that fever is caused by the inhibition of hypothermic MnPO^PACAP^ neurons, and to better understand the synaptic targets by which MnPO^PACAP^ neurons produce a febrile response, we chose optogenetic inhibition with Archaerhodopsin-T (ArchT). *Adcyap1*^*Cre*^ male mice were injected with an AAV-DIO-ArchT:GFP in the MnPO causing Cre-dependent expression of a light-responsive proton pump that results in hyperpolarization of neurons^13^. Freely behaving mice implanted with telemetric Tb sensors were kept at an ambient temperature of 22 ± 2 °C. After at least 48 h of habituation, we optogenetically inhibited either MnPO^PACAP^ cell bodies (Fig 1q–r) or their synaptic terminals in the DMH or RPa (60 s of laser-on followed by 30 s of laser-off for 15 min) (S Fig 2a–e). This protocol induced an elevation of Tb by 0.5–0.6 °C that lasted >2 h after the termination of opto-inhibition of the cell bodies or their terminals in the DMH (Fig S2d–e). The prolonged elevation of Tb after inhibition MnPO^PACAP^ cell bodies or their terminals in the DMH is unlikely to be due to thermal inertia, as the inhibition of MnPO^PACAP^ terminals in the RPa caused a similar elevation of Tb, but of much shorter duration. This suggests that even after only 15 min of intermittent inhibition of MnPO^PACAP^ terminals in the DMH, there is a long-lasting increase in neural circuit activation that supports elevation of Tb.

## Activation of EP3R-expressing MnPO neurons produces a reversible state of deep hypothermia

Activation of glutamatergic (but not GABAergic) neurons in the MnPO in mice produces deep hypothermia, mimicking a torpor or hibernation-like state^1–4^. Nearly all MnPO^EP3R^ neurons (>96%) express Vglut2 in mice^11^ and a large percentage (60–75%) co-express PACAP or QRFP. We hypothesized that the expression of EP3R would identify a key population of preoptic glutamatergic neurons that express PACAP or QRFP and are necessary to cause torpor responses.

To test this hypothesis, we injected AAV-DIO-hM3Dq:mCherry (hM3Dq) into the MnPO of *Ptger3*^*Cre*^ male mice to permit selective activation of the EP3R-expressing MnPO neurons (Fig 2 a–d) while evaluating changes in Tb. After 4 weeks to allow hM3Dq expression, mice received an injection of either saline or a ligand for the hM3Dq receptor, clozapine-N-Oxide (CNO) or deschloroclozapine (DCZ). About 1 h after CNO (0.3 mg/kg), the mice kept at room temperature (22 ± 2°C) entered a deeply hypothermic state (Tb dropped from about 36 °C to 24–27 °C) that lasted from 24 to >72 h (Fig 2e). In addition, we found a robust reduction of the locomotor activity (LMA), heart rate (HR), energy expenditure (EE) and food intake during the same period (Fig 2f–g and Fig S 4a), but no changes in body weight (BW) were observed (Fig 2h and Fig S 4b).

Activation of neurons by hM3Dq typically lasts about 3–4 h after CNO injection, suggesting that the long duration of hypothermia caused by activation of the MnPO^EP3R^ neurons was due to a prolonged effect on activity of the MnPO^EP3R^ neurons or their post-synaptic targets, the converse of the prolonged elevation of Tb that we saw after brief inhibition of MnPO^PACAP^ neurons. However, we were concerned that the hypometabolism during torpor may prolong the presence of CNO in the blood of the hypothermic animals. Hence, we used DCZ as a selective ligand for hM3Dq that has a half-life of 1 h in the plasma of animals^14,15^. We were surprised to find that treatment either with either DCZ or CNO at the dose of 0.1 mg/kg produced an equally potent and long-lasting period of hypothermia, hypoactivity and bradycardia in mice (Fig S 3g). Howwever, the long-lasting effect of the drugs could have been due to reduced metabolism of CNO caused by hypothermia (Fig 2f and Fig S 4a). To test this, we rewarmed mice expressing hM3Dq in MnPO^EP3R^ neurons during CNO-induced hypothermia, returning their Tb to 37° C for 12 h during the dark period (Fig S 4c). This rewarming resulted in a transient partial return of Tb, LMA and HR toward normal, but when we moved the animals back to a 22° C environment, their Tb LMA and HR fell back to levels typically seen 12–24 h after CNO injection in hypothermic animals. As we could not be sure that we had restored metabolism of drugs like CNO during rewarming, we measured CNO levels in the blood of the hypothermic mice 12–24 h after CNO injection. Mass spectrometry showed that CNO was readily detected in the plasma of a mouse 1 h after injection, but was no longer detectable in the plasma of hypothermic mice 12–24 h after the drug injection (n=3). Thus, the long-lasting hypothermia does not appear to be due to the ligand for the hM3Dq receptor persisting in the plasma and is likely to be a consequence of prolonged activation of the neurons that cause hypothermia or their downstream targets, such as the DMH.

**Fig 3.**
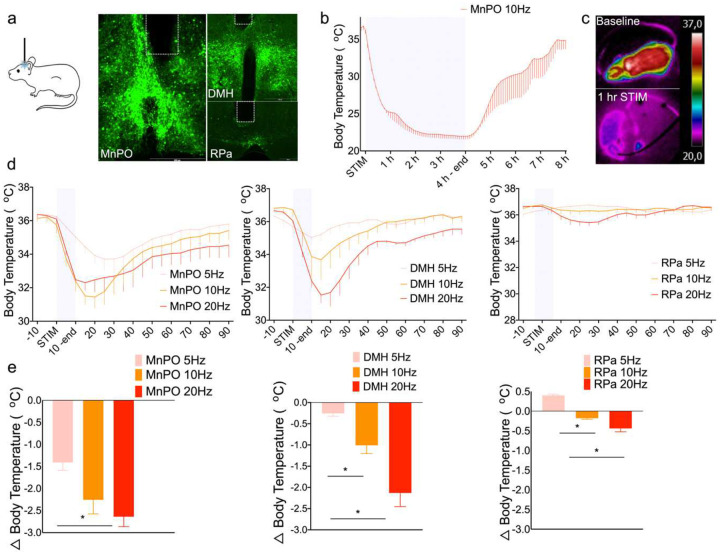
Optogenetic activation of EP3R-expressing MnPO neurons produces torpor-like bouts. A schematic figure of the protocol for optogenetic stimulation experiments in the left panel and a representative image of AAV-DIO-ChR2:YFP expression in the MnPO cell-bodies and their terminals in the DMH and RPa in the right panel of a *Ptger3*^Cre^-cre mouse (a). Prolonged optogenetic stimulation of MnPO^EP3R^ neurons (10 Hz, 2 s on, 3 s off, n=4) for 4 h reduced Tb from a baseline of 36.59 ± 0.4°C to a nadir of 22 ± 0.4°C. Tb recovered slowly over the subsequent 4 hr without stimulation 34.83 ± 1.2 °C, but was still about 1.8 °C lower them their baseline (b). Near the nadir, surface temperature of the entire animal was near ambient temperature (c). Optogenetic stimulation of MnPO^EP3R^ neurons for 5 sec each min for 10 min (n=4) produced a steeper fall in Tb and slower recovery at 10 and 20 Hz than at 5 Hz (mean change in Tb compared to prestimulation baseline during the 90-min period was −1.4 ± 0.17°C MnPO Stim 5 Hz vs −2.2 ± 0.32°C MnPO Stim 10 Hz vs −2.6 ± 0.22°C MnPO Stim 20 Hz, MnPO Stim 5 Hz vs MnPO Stim 10 Hz, F(2,54)=2.4, One-way ANOVA, followed by Bonferroni’s post hoc test, p=0.003 5 Hz vs. 20 Hz, d,e). The frequency dependence was even more pronounced during stimulation of the MnPO^EP3R^ synaptic terminals in the DMH (n=3), where 20 Hz stimulation reduced Tb to the same levels as stimulation of the cell bodies with a similar slow recovery (mean change in Tb over the 90-min period was −0.2 ± 0.06°C DMH Stim 5 Hz vs −1.0 ± 0.19°C DMH Stim 10 Hz vs −2.1 ± 0.32°C DMH Stim 20 Hz, DMH Stim 5 Hz vs DMH STIM 10 Hz, F_(2,54)_=8.3, One-way ANOVA, followed by Bonferroni’s post hoc test, p=0.002 and DMH STIM 5 Hz vs DMH STIM 20 Hz, F(2,54)=8.3, One-way ANOVA, followed by Bonferroni’s post hoc test, n=3, p<0.0001 5 Hz vs. 20 Hz, p=0.007 5 Hz vs. 20 Hz and p=0.003 10 Hz vs. 20 Hz). The 10–20 Hz brief stimulation produced a fall in Tb similar to a naturally occurring torpor bout due to food deprivation (d-e). Optogentic stimulation of the MnPO^EP3R^ terminals in the RPa (n=4) did not cause a statistically significant change in Tb.

**Fig 4.**
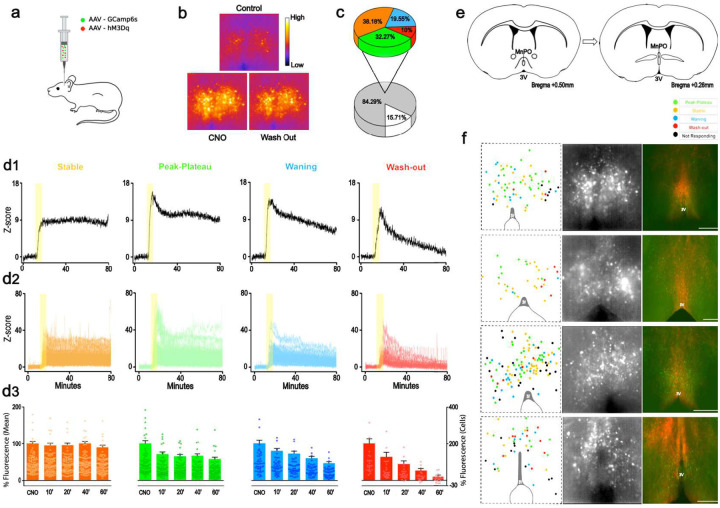
EP3R-expressing MnPO neurons display prolonged activation after brief chemogenetic stimulation. AAV-DIO-GCaMP6s and AAV-DIO-hM3Dq-mCherry were injected into the MnPO of *Ptger3*^Cre^ mice for *in vitro* GCaMP-based calcium imaging recordings of EP3R MnPO neurons *in vitro* during and after chemoactivation (a). Time lapse of pseudocolored fluorescence images showing intensity of activation of MnPO^EP3R^ neurons during application of CNO compared to baseline (control), and then 60 min after the CNO was washed out. Images acquired at: 10 min in control, 5 min in CNO (0.5 μM) and 60 min in wash-out (b). Out of 261 neurons that were recorded, 84% showed increased activity in response to CNO, and these neurons showed four patterns of response (c): 38% of neurons had a rapid increase in activity that then was stable over the next hour after washout of CNO (orange); 32% reached a peak and then during the washout had a small reduction in fluorescence to a stable level over the next hour (green); 20% peaked and then had a gradual slowly waning response (blue) and 10% peaked then had a more rapid decline in fluorescence (red) after CNO was removed. The overall responses of these populations are shown in d1; the response of individual neurons in each group in d2; and the mean response at different time points during the experiment in d3. Rostral to caudal anatomical levels where the recordings were conducted is shown in e, and the distribution of neurons of each type (color coding as in d) activated by is shown in the *left column*, GCaMP fluorescence in the recording slices in the *center column* and GCaMP (green) and hM3Dq-mCherry (red) native fluorescence in the fixed recorded slices in the *right column* (scale bars: 500 μm).

To better appreciate the duration of stimulation required to produce such prolonged hypothermia, we used optogenetic stimulation of the MnPO^EP3R^ neurons (Fig 3a). We found that prolonged (10 Hz for 2 s on and 3 s off, for 4 h) photostimulation of MnPO^EP3R^ neurons reproduced the hypothermic effect found after chemogenetic activation of MnPO^EP3R^ neurons. During a 4-h photostimulation period, in mice held at 22 °C the Tb fell from 36.6 ± 0.4°C to 22 ± 0.4 °C, n=4. After photostimulation ended, the mice gradually rewarmed but even 4 h later they still had a Tb less than baseline, 34.8 ± 1.2 °C (Fig 4b). The mechanism causing hypothermia was explored by infrared image analyses. We found tail artery vasodilation and strong suppression of BAT thermogenesis during the optogenetic or chemogenetic stimulation (Fig 4c and S Fig 3h). In addition, we found that short-term optogenetic activation of the MnPO^EP3R^ neurons at 10–20 Hz for 5 seconds each minute for 10 min initiated a torpor bout that lasted for ~1–2 h (Fig 4d–e). This result suggests that even relatively brief stimulation of MnPO^EP3R^ neurons produces a long-lasting effect on Tb.

We explored the downstream sites that allow the drop in Tb caused by optogenetic stimulation of the MnPO^EP3R^ neurons or their synaptic terminals in the DMH or RPa (n=3) for 5 s each min for 10 min. Stimulation of the cell bodies or the terminals in the DMH at 10 or 20 Hz produced a torpor-like response. This response was not produced by optogenetic stimulation of the MnPO^EP3R^ terminals in the RPa (Fig 4 d–e), which caused only a small drop in Tb (n=4). Hence the bulk of the prolonged hypothermic response is due either to prolonged activation of MnPO^EP3R^ neurons, their targets in the DMH or further downstream (Fig S6).

## EP3R-expressing MnPO neurons remain highly active after stimulation ceases

To explore whether MnPO^EP3R^ neurons themselves show prolonged activation after brief stimulation, we measured changes in intracellular calcium signaling in individual MnPO^EP3R^ neurons in preoptic slices after brief stimulation.

The MnPO of *Ptger3*^*Cre*^ mice was injected with AAV-DIO-GCaMP6s and AAV-DIO-hM3Dq-mCherry (Fig 4a). Three to four weeks after the AAV injections, we recorded GCaMP fluorescent activity from individual EP3R-expressing neurons in hypothalamic slices. Brief applications of CNO (0.5 μM for 5 min) increased GCaMP fluorescence in 85% of the recorded neurons. The calcium fluorescence response was rapid and, in most neurons, persisted over an hour after the wash-out of CNO (Fig. 4b). Further analyses revealed four types of response patterns: after the initial activation by CNO approximately 40% of the MnPO^EP3R^ remained stably active for >1 h after the wash-out of CNO (stable cells); 30% showed an initial peak in fluorescence followed by a sustained response (peak-plateau cells); 20% showed a slow and incomplete gradual return to baseline fluorescent levels (waning cells) and 10% fully returned to baseline fluorescence levels within 60 min of CNO wash-out (wash-out cells) (Fig 4c–d). Neurons displaying different classes of responses showed distinctive anatomical distributions in the MnPO region (Fig 4e–f), with the neurons that maintain a high level of intracellular calcium after a short activation (stable and peak-plateau cells) located mainly in the rostral-ventral portion of the MnPO. These results suggest that at least part of the mechanism underlying the ability of MnPO^EP3R^ neurons to produce long-lasting hypothermia is that these neurons continue to show high levels of activity as measured by intracellular calcium levels for long periods after the stimulus is removed. However, it is also possible that neurons in DMH or further downstream that are activated by MnPO^EP3R^ neurons may have similar responses. If so, the cascade of prolonged responses in the pathway may contribute to the long-duration hypothermia seen after activation of MnPO^EP3R^ neurons by a single dose of CNO or DCZ.

## MnPO^EP3R^ neurons work as a master switch of the thermoregulatory and metabolic system

**Fig 5.**
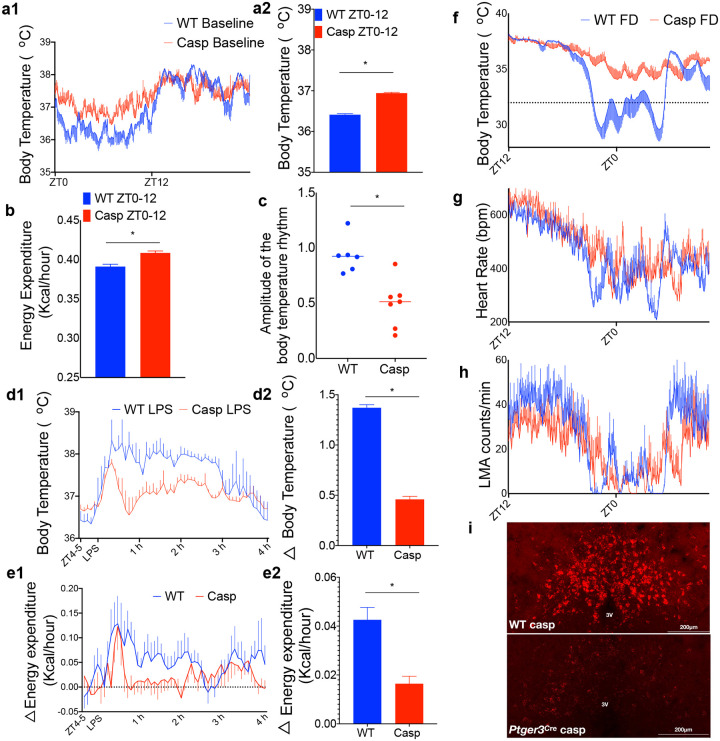
EP3R-expressing MnPO neurons work as a two-way switch. Deletion of MnPO^EP3R^ neurons by injection of AAV-DIO-caspase3 in the MnPO of male and female *Ptger3*^Cre^ mice elevated baseline Tb and reduced EE during the dark (sleep) phase but not the light (wake) phase compared to WT littermates (ZT0-ZT11; Tb 36.41 ± 0.02 °C WT vs. 36.94 ± 0.02 °C Casp, unpaired t-test p<0.0001, n=6 and 7 mice, respectively (4 and 3 female mice in each group, respectively); EE 0.39 ± 0.002 kcal/hour WT vs 0.41 ± 0.002 kcal/hour, unpaired t-test p<0.0001, n=6 and 5, respectively) (a and b). This effect is also reflected in a smaller amplitude of the 24 h diurnal rhythm of Tb in EP3R-deleted mice (difference of mean Tb during the dark vs light periods was 0.93 ± 0.06 °C in WT littermates and 0.49 ± 0.08 °C in EP3R-deleted mice, unpaired t-test p=0.001 n=6 and 7, respectively) (c). After LPS injection, male mice depleted of MnPO^EP3R^ neurons showed typical stress hyperthermia and elevated EE for about 45 min due to the handling. However, 1–3 h after the LPS injection, the Tb and EE of MnPO^EP3R^-deleted mice were nearly the same as prior to the LPS injection, while WT mice showed a typical fever response (1.37 ± 0.03 °C WT LPS vs 0.46 ± 0.02 °C Casp LPS, unpaired t-test p<0.0001, n=5 and 6, respectively) and elevation of EE (0.04 ± 0.005 kcal/hour WT LPS vs 0.01 ± 0.003 kcal/hour Casp LPS, unpaired t-test p<0.0001, n=7 and 5, respectively) (d-e). In response to food deprivation beginning at ZT12, intact WT mice but not MnPO^EP3R^-deleted mice showed periods of torpor (AUC of the Tb 799.7 ± 25.19 SD in WT mice vs 461.3 ± 16.3 SD in AAV-Casp treated mice, p=0.002, n=6 and 7, respectively) (f), although the Casp-FD mice showed an ~2 °C gradual fall in Tb instead of the usual ~ 1°C rise during the active period in WT mice fed *ad libitum*. Deletion of MnPO^EP3R^ neurons slightly reduced the fall in HR (371.7 ± 6.7 WT bpm vs. 398.0 ± 4.87, p=0.001, n=5 and 4, respectively), but not overall LMA (−16.29 ± 1.4 LMA WT ZT0-ZT12 vs −13.28 ± 0.95 LMA ZT0-ZT12 Casp, unpaired t-test p=0.08, n=6 each group) seen with food deprivation relative to the baseline at ZT12 (g-h). After injection of AAV-DIO-casp in the MnPO, we confirmed the specificity of deletion of Cre-expressing neurons by using RNA-scope in situ hybridization. We found intense expression of *Ptger3* in WT mice and very few remaining neurons expressing mRNA for EP3R in the MnPO of *Ptger3*^Cre^ mice (n=2) (i).

We then tested the necessity of MnPO^EP3R^ neurons in modulating Tb and metabolic rate (MR) in natural conditions. For these experiments, we injected pAAV-DIO-taCasp3-TEVp (AAV-Casp) in the MnPO of *Ptger3*^*Cre*^ mice and their WT littermates. We found that the baseline Tb of the MnPO^EP3R^-deleted mice (Casp) was inappropriately high compared to the WT control mice during the light (inactive) phase (ZT0-ZT11) in both male and female mice (36.6 °C ± 0.02 female WT vs. 36.1 °C ± 0.03 male WT vs. 37.0 °C ± 0.03 female Casp vs. 36.8 °C ± 0.02 male Casp, Kruskal-Wallis test, followed by Dunn’s post hoc test, p<0.001, n=4,2,4,3 respectively) (Fig 5a), suggesting that the MnPO^EP3R^ neurons play a key, although probably not exclusive role in reducing Tb during the inactive phase in male and female mice, when the animals are largely sleeping (ZT0-ZT11; Tb 36.41 ± 0.02 °C WT vs. 36.94 ± 0.02 °C Casp, unpaired t-test p<0.0001, n=6 and 7 mice, respectively). This effect was also reflected in a smaller amplitude of the 24-h diurnal rhythm of Tb in MnPO^EP3R^-deleted mice (Fig 5b). In addition, MnPO^EP3R^-ablated mice showed a consistently higher Tb compared to littermate control mice during the light phase even when exposed to a cold (4 °C) environment (Fig S 5d).

To test the ability of the MnPO^EP3R^-ablated mice to produce fever and metabolic adaptive responses associated with inflammatory or infectious conditions, we injected them with LPS. Similar to the mice with deletion of EP3R from PACAP neurons, mice with deletion of MnPO^EP3R^ neurons showed a typical elevation of their Tb and energy expenditure (EE) above baseline during the first 30–45 min after LPS injection – a hyperthermic effect caused by the stress of handling. However, 1–3 h after the LPS injection, the Tb and EE of MnPO^EP3R^-deleted mice were close to baseline levels (n=5), while WT mice (n=5) showed a typical fever response (an increase of Tb of about 1.4 °C) and elevated EE by about 0.04 Kcal/h (Fig 5d–e). In three out of five *Ptger3*^*Cre*^ mice, in which the injections successfully targeted the core and wings of the MnPO region, but not the ventral lateral preoptic (VLPO) region where a cluster of neurons express EP3R, we observed a hypothermic response (Δmax below baseline of −0.8 °C ± 0.4 SEM, n=3) following the initial stress hyperthermia. This response is similar to the effect of LPS after deletion of EP3R from the CNS or deletion of EP3R from all glutamatergic (Vglut2) neurons^9,11^. After this hypothermia, these three mice showed a small increase of Tb to 0.3 °C ± 0.03 SEM above baseline, while WT mice had fever of 1.4 °C ± 0.03 SEM (n=5) during the 1–3 h after LPS injection (Fig 5d and S Fig 5e). Two other *Ptger3*^*Cre*^ mice in which the AAV-Casp injection included mainly the dorsal MnPO showed a fever response to LPS injection that was about 50% (0.7 °C ± 0.03 SEM, n=2) of the WT fever response. We also tested the thermoregulatory responses of these mice during an immune reaction caused by a synthetic double-stranded RNA (polyinosinic-polycytidylic acid, poly I:C)^16^ and found that MnPO^EP3R^-deletion only partially attenuated fever caused by this antigen even in the mice with the most successful injection placement that showed hypothermia after LPS. Hence, poly I:C fever has a component that is not dependent upon the MnPO^EP3R^ neurons (S Fig 5c).

In response to food deprivation (FD) beginning at lights out (ZT12), intact WT male and female mice showed a gradual fall in Tb starting around four hours before the light phase (mean Tb ZT20, 35.4 °C ± 0.6 SD female WT vs. 36.2 °C ± 0.4 SD male WT) followed by torpor episodes beginning around ZT21 (a drop of Tb below 33 °C associated with a hypometabolic state to conserve energy) (Fig S 5b), as well as reduction of LMA and HR (Fig 5 f–h). Mice with deletion of MnPO^EP3R^ neurons showed a gradual fall in Tb (mean Tb ZT20, 36.8 °C ± 0.4 SD female casp vs. 35.9 °C ± 0.3 SD male casp) but from ZT21 onward failed to produce torpor responses (mean Tb of 34.3 °C ± 1.1 SEM at ZT0; n=5; Fig 5f). Two *Ptger3*^*Cre*^ mice in which the AAV-Casp injection included only a dorsal part of the MnPO showed more torpor bouts and lower mean Tb during the same period (33.6°C ± 1.7 SEM at ZT0, n=2). WT mice subjected to the same FD protocol (n=6) showed an average Tb of 31.2°C ± 1.7 SEM at ZT0 (n=5). Thus, the torpor bouts appear to depend critically upon the MnPO^EP3R^ neurons, particularly those in the ventromedial part of the MnPO, and the bouts do not occur without their participation.

## Discussion

Our findings demonstrate that the preoptic neurons that cause hypothermia when stimulated and those that cause fever when inhibited constitute a single population that is characterized by expression of the EP3R. Upton and colleagues^8^ compared the expression of various genes related to thermoregulatory control among the neuronal expression types identified by Moffitt et al. in their atlas of preoptic cell types^17^, and by Hrvatin et al. in examining neurons that showed cFos expression during torpor using a Fos-trap method^1^. They pointed out that the e13 cell type identified by Moffitt et al. has relatively high levels of expression of mRNA for *Adcyap1*, *Esr1*, and *Opn5*^17^, all of which had been previously shown to be expressed in preoptic neurons that drive hypothermia^1–3,7,17,18^. The e13 cell type also had the highest expression of *Ptger3* mRNA. The e10 cell type identified by Hrvatin et al. had an almost identical pattern of mRNA expression for these markers, indicating that it is probably the same cell type. Oddly, though, whereas the e10 cell type of Hrvatin et al. also expressed the *Qrfp* mRNA that Takahashi et al.^3^ had identified as a marker for hypothermic preoptic neurons, the e13 cell group of Moffitt et al. did not. On the other hand, the e7 group of Moffit and colleagues expressed a somewhat lower level of *Ptger3* but high levels of *Qfrp* mRNA. Other minor differences, e.g., in expression of Transient receptor potential (*Trp*) mRNAs associated with intrinsic thermosensitivity, suggest that the two studies have identified overlapping, but not necessarily identical neuronal populations. Thus, there may be some heterogeneity in gene expression among the neurons that participate in hypothermia/torpor. However, our studies indicate that the MnPO^EP3R^ neurons uniquely identified the population of preoptic neurons that cause torpor due to insufficient energy stores and fever due to prostaglandin release during inflammation. These results support our hypothesis [cite Nature commentary here] that the MnPO^EP3R^ population constitutes a two-way switch allowing animals to adapt body temperature to threatening conditions (allostatic threat). The ability of animals in which the MnPO^EP3R^ neurons were ablated to maintain a Tb of 37° C during the light cycle even in a 4° C environment indicates that they are not required for cold defense. Their role in responding to homeostatic regulation of Tb under warm conditions remains a subject for further study.

Other populations of neurons such as those in the ventromedial preoptic area adjacent to the MnPO show increased Fos expression during LPS fever^16,19^, but it is not clear whether the neuronal activation caused or was a consequence of the LPS fever. Unlike the MnPO^EP3R^ neurons which are glutamatergic^11^, the ventromedial preoptic neurons that express Fos during LPS fever are GABAergic and therefore belong to a different cell population that apparently is not capable of causing LPS fever in the absence of EP3R in the MnPO. Deletion of the ventromedial preoptic neurons reduced LPS fever (but LPS did not cause hypothermia), so they did not have as potent an effect on LPS fever as did deletion of the MnPO^EP3R^ neurons (after which LPS caused hypothermia). Thus, the ventromedial preoptic neurons are apparently part of the downstream pathways activated during LPS fever, but the elevation of Tb depends upon PGE2 action on the MnPO^EP3R^ neurons.

Based on our previous results together with the work from other labs^1,3^, we hypothesized that MnPO^EP3R^ neurons in mice, which are essential for causing LPS fever when inhibited by the PGE2, might also be important for the production of hypothermia when activated^20^. We therefore monitored changes in Tb, LMA, HR, EE and food intake of mice while chemogentically activating MnPO^EP3R^ neurons. In addition to hypothermia, mice showed profound reduction in physical activity and energy expenditure as well as bradycardia. This response lasted >72 h in some mice, even though the CNO that triggered the response was absent in the serum by 12 h. The persistent hypothermic response induced by stimulation of MnPO^EP3R^ neurons was reproduced by brief optogenetic activation of MnPO^EP3R^ neurons or their terminals in the DMH for 10 min, which caused hypothermia lasting >2 h.

To explore whether the prolonged hypothermia might reflect persistent activation of the MnPO^EP3R^ neurons once turned on, we examined GCaMP fluorescence signal in these neurons in a slice preparation. Most of the MnPO^EP3R^ neurons showed prolonged increases in intracellular calcium for more than an hour after the CNO stimulus was removed. Whether there is a similar prolonged neuronal activation and enhancement of intracellular calcium in downstream neurons, e.g., in the DMH, remains to be studied. In addition, it is not known whether the prolonged activation of MnPO^EP3R^ neurons represents a cell-autonomous response to brief stimulation or may be due to recurrent excitation via a circuit within the immediate vicinity (in the slice). This question will have to be resolved with intracellular recordings from the MnPO^EP3R^ neurons after brief stimulation and inhibition.

In a final test of the role of the MnPO^EP3R^ neurons as a two-way switch, we examined their necessity for generating torpor, as well as their previously demonstrated necessity for producing LPS or prostaglandin E2 fever^9,11^. Previous studies silencing either PACAP-expressing or QRFP-expressing preoptic neurons found that they did not prevent hypothermia in response to food deprivation in mice (Fig 4, Hrvatin et al., 2020 and Extended Fig 10, Takahashi et al., 2020)^1,3^. By contrast, deletion of the MnPO^EP3R^ neurons that was sufficient to abrogate the hyperthermic (fever) response to LPS also almost completely prevented the hypothermic (torpor) response to food deprivation at 22°C. These observations indicate the MnPO^EP3R^ neurons are the core population that acts as a two-way switch, responsible both for hyperthermic (LPS fever) and hypothermic (torpor) regulation.

The role of MnPO^EP3R^ neurons in homeostatic and circadian thermoregulation remains to be explored. However, our finding that the baseline Tb is elevated during the light (sleep) cycle but not the dark (active) cycle in MnPO^EP3R^-deleted mice suggests that these neurons play a role in circadian regulation of Tb. The role of MnPO^EP3R^ neurons in homeostatic warm and cold defense is less clear. Acute inactivation of the MnPO^EP3R^ neurons causes a roughly 1–1.5° C rise in Tb^21^, which would be consistent with their playing a role in cold defense by being tonically inhibited at an ambient temperature of 22 °C. However, to prevent counter-regulatory responses, their inhibition would also have to disable other warm defense circuits. Conversely, exposing animals with deletion of MnPO^EP3R^ neurons to a 4 °C environment does not reduce their Tb, so there must be other cold defense circuits that do not depend upon MnPO^EP3R^ neurons. On the other hand, chemogenetic activation of the MnPO^EP3R^ neurons causes Tb to fall to just above ambient temperature at 22 °C, and to come back up to 38 °C when placed in a 37 °C environment. This essentially poikilothermic response when MnPO^EP3R^ neurons are activated, suggests that they must be capable of disabling both other warm and cold defense mechanisms. These hypotheses will have to be tested by further experimentation.

We expect that the MnPO^EP3R^ thermoregulatory neurons are conserved across species, as drugs that block prostaglandin production (e.g., aspirin, acetaminophen, ibuprofen) reduce or prevent inflammatory fever in all mammalian species tested, including humans. In addition, even species that do not normally show torpor or hibernation, such as rats or non-human primates, can show hypothermic responses to activation of preoptic neurons^3,22^, and a recent paper found that specific activation of MnPO^EP3R^ neurons in rats can also cause hypothermia^21^. Humans who have had preoptic injuries also can have episodes of paroxysmal hypothermia, resembling torpor^23^. Hence, it will be important to study whether other mammals, including humans, share this same genetic marker and brain circuits to regulate the thermoregulatory system. If so, it may be possible to harness activation of these pathways to modulate Tb for therapeutic proposes^24^ in humans.

## Methods

### Animals

All animal care and experimental procedures were approved by the Beth Israel Deaconess Medical Center Institutional Animal Care and Use Committee. We used *Ptger3*^*Cre*^ mice which were generated by Dr. Richard Palmiter (B6.Cg-*Ptger3*^*tm1.1(cre/GFP)Rpa*^, The Jackson Laboratory Stock No: 035575 and validated in our laboratory (Fig S1) and the subtype *Ptger3*^*flox/flox*^ mice (B6; 129-*Ptger3*^*tm1Csml*^/J; Stock No:008349, Jackson laboratories, MA) originally produced in our laboratory that have loxP sites flanking exon 1 of the *Ptger3* gene. We also used *Adcyap1*^*Cre*^ mice that were generated by Dr. Bradford Lowell. *Adcyap1*^*Cre*^*Ptger3*^*flox/flox*^ (PACAP+EP3R-null mice) mice were generated by crossing *Ptger3* homozygous flox mice with *Adcyap1*^*Cre*^ mice^11,25^ and the resultant offspring that are heterozygous for *Ptger3*^*flox/flox*^ and *Adcyap1*^*Cre*^ were again crossed with *Ptger3* homozygous flox mice to get *Adcyap1*^*Cre*^*Ptger3*^*flox/flox*^ (hereafter referred to as PACAP+EP3R-null mice) mice, in which exon 1 of both copies of the *Ptger3* gene is deleted in the PACAP+ neurons. We also used *Adcyap1*^*Cre*^ -GFP (*Adcyap1*^*Cre*^; *Rosa26*^*LSL-L10:GFP*^ mice), and Vglut2-IRES-Cre-GFP; Vglut2-IRES-Cre; *Slc17a6*-IRES-Cre::R26-loxSTOPlox-L10-GFP)^11,25,26^.

The age of mice at the time of experimentation ranged between 12–22 weeks. We used male and female mice, as indicated in the text. Mice were individually housed in standard plastic cages with standard corn cob bedding with nesting materials on a 12 h light: 12 h dark cycle at ambient temperatures ranging between 22 ± 2°C. Mouse chow (Teklad F6 Rodent Diet 8664) and water were provided *ad libitum*, unless mice were exposed to 24 h of food deprivation, as specified below.

### Surgery

All surgeries were performed in sterile conditions. Mice were anesthetized with the mix of ketamine/xylazine (100 and 10 mg/kg, respectively, i.p.) with additional doses of 10% of the initial dose throughout surgery to eliminate the withdrawal reflex. Stereotaxic microinjections or optical fiber placements were made into the MnPO (coordinates: AP = +0.43 mm, L = 0.0, DV = −4.6 mm from Bregma), DHA/DMH (coordinates: AP = −1.85 mm, L = 0.3, DV = −4.45 mm from Bregma) and RPa (coordinates: AP = −2.2 mm, L = 0.0, DV = −5.5 mm from Lambda)

Mice were implanted with a radiotelemetry sensors (TA-F10 or ETA-F10, DSI) in the intra peritoneal space via laparotomy. For the electrocardiogram (HR) recordings, we fixed one the electrodes attached to the ETA-F10 sensor on the chest muscles and the second electrode in the abdominal region. Then, the muscle layers around the electrodes were closed with absorbable sutures and the abdominal wall was sutured using absorbable sutures. Meloxicam treatment, for analgesia, was administered prior to surgery. Mice were allowed to recover at least 10 days prior to experimentation. Following recovery, mice showed no signs of discomfort and gained weight normally.

### Viral Vectors

AAV1-DIO-GCAMP6 (packaged and obtained by Addgene) was delivered into the MnPO of each mouse by a single brain injection of 90–120 nL.

AAV8-CAG-DIO-ArchT-GFP that co-expresses the Archaerhodopsin TP009 T (ArchT) in a Cre dependent manner. This virus vector was procured from the University of North Carolina vector core (UNC). Injections were made using the volume of 30–90 nL^13^.

AAV8-hSyn-DIO-hM3D(Gq)-mCherry (AAV-hM3Dq) produced at the University of North Carolina virus core was used in the volume of 60–90 nL^13^.

pAAV5-EF1a-DIO-hChR2(H134R)-EYFP-WPRE-hGHpA (AAV5), *AAV-ChR2-eyfp*. It was delivered into the MnPO of each mouse by a single brain injection of 90–150 nL.

pAAV5-DIO-taCasp3-TEVp (AAV-Casp) was packaged and obtained by Addgene. It was delivered into the MnPO of each mouse by a single brain injection of 120–150 nL. In some cases, a mix of AAV-DIO-casp and non-cre dependent AAV-GFP in the proportion of 1:1 to label the injection sites (Fig S5 e).

### Intraperitoneal injections

Lipopolysaccharide from Escherichia coli 0111:B4 (LPS), (Sigma catalog #L2630) was used at dose of 0.02 mg/kg, and compared to saline. LPS injections were performed between 11:00 and 13:00 pm^11^.

Polyinosinic-polycytidylic acid (poly I:C) (Sigma catalog 42424-50-0) was used at dose of 10 mg/kg, and compared to saline. The injections were performed between 11:00am and 13:00pm.

Clozapine-N-oxide (CNO), (Sigma catalog #34233-69-7), 0.1mg/kg or 0.3mg/kg, was injected between 10:00 and 12:00pm^13^.

Deschloroclozapine (DCZ), MedChemExpress catalog #1977-07-7), 0.1mg/kg, was injected between 10:00 and 12:00pm^15^.

### Body temperature (Tb), locomotor activity (LMA) and heart rate (HR) recordings

Tb, LMA and HR was recorded using the radiotelemetry DSI system (sensors ETA-F10). The signal was sent from the telemetry sensors previously implanted in mice to DSI receivers and converted using the PhysioTel HD and PhysioTel (DSI) hardware. We used the mean of core temperature, locomotor activity and heart rate every 5 min.

### Food Deprivation

Mice were kept at 22 ± 2 °C were food deprived for 24h starting 0–1 h before the beginning of the dark phase (ZT12). Water was provided *ad libitum*.

#### Energy expenditure measurements:

We used the Promethion metabolic cages by Sable Systems International to measure oxygen consumption (VO2), carbon dioxide production (VCO_2_) and respiratory exchange ratio (RER) every 3–5 minutes. These cages were maintained at 23 +/− 1 °C and we used a range of 4–37 °C environmental temperature for experiments described in the text or legends. Food consumption and body mass measurements were constantly measured as well. For the FD, we used a preprogrammed timing of fasting, and refeeding for the 24h food deprivation.

### Fiber photometry in vivo

The basic setup for fiber photometry consists of the LED light source(s) that can provide microWatts (μW) of fluorescence excitation light (405nm and 460nm) and a photo-detector(s) sensitive to picoWatts (pW) of fluorescence emission, all packaged within compact Fluorescence Mini Cube (FMC, Doric lenses, Quebec, Canada) with appropriate spectral filtering of excitation and emission light. Animals were connected with a low auto-fluorescence fiber-optic patch cord with a chronically implanted fiber-optic cannula. Briefly, light of two wavelengths was transmitted by the LED’s (460nm and 405 nm isobestic) were directed down a fiber optic patch cord through the pre-implanted fiber optic cannula (Doric lenses #B280-4419-5) in the MnPO of each mouse. Emitted gCaMP6s fluorescence was detected by the photodetectors in the FMC and converted to a voltage signal which was acquired using the Spike2 software. Post-hoc analysis of the acquired data was also performed using Spike2 analysis that involved low pass filtering of acquired signal, normalization of the data to the baseline or control recordings (isobestic signal).

### Calcium signaling in vitro

*Ptger3*^Cre^ mice (*n*=3) were injected into the MnPO with a mix of AAV-GCAMP6s and AAV-hM3Dq. Three to four weeks after the AAV injections, mice were euthanized for *in vitro* calcium imaging recordings. Mice were first anesthetized with isoflurane (5% by inhalation) then transcardially perfused with an iced-cold NMDG-based solution containing in mM: NMDG 92, KCl 2.5, HEPES 20, Glucose 25, NaHCO_3_ 30, NaH_2_PO_4_ 1.25, CaCl_2_ 0.5, Thiourea 2, Na-Ascorbate 5, Na-Pyruvate 3, MgSO_4_ 10, N-acetyl-L-cysteine 6 (95% O_2_ / 5% CO_2_; pH 7.24–7.3; 300 mOsm). Following decapitation, the mouse brains were rapidly removed and sectioned in coronal slices (250-μm thick) in ice-cold NMDG–based solution using a vibrating microtome (VT1200S, Leica, Bannockburn, IL). Slices containing the MnPO were first incubated at 35°C for 10 minutes and then transferred in a HEPES-based solution for at least 1 hour (room temperature) before start recording^27,28^. The HEPES-based solution contains in mM: NaCl 92, KCl 2.5, HEPES 20, Glucose 25, NaHCO_3_ 30, NaH_2_PO_4_ 1.25, CaCl_2_ 2, Thiourea 2, Na-Ascorbate 5, Na-Pyruvate 3, MgSO_4_ 2, N-acetyl-L-cysteine 6 (95% O_2_ / 5% CO_2_; pH 7.24–7.3; 330 mOsm) Calcium imaging recordings of the MnPO^EP3R^ neurons expressing GCaMP6s were conducted using a fixed stage upright microscope (BX51WI, Olympus) equipped with a water immersion lens (Olympus 10X / 0.8 NAW) a fluorescence filter set for GFP and a CMOS camera (Thorlabs, Newton, NJ). Brain slices were recorded submerged and perfused (1–2 ml/min) with a Na-based solution containing in mM: NaCl 125, KCl 2.5, Glucose 20, NaH_2_PO_4_ 1.25, CaCl_2_ 2 and MgCl_2_ 1.3 (95% O_2_ / 5% CO_2_; pH 7.24–7.3). Blue light pulses for GCamp6s stimulation were provided from a 5W Luxeon blue light-emitting diode (470-nm wavelength; #M470L2-C4; Thorlabs). To reduce the light exposure time the LED was only turned-on during image acquisition using a custom-made Python script which controls both the LED and the CMOS camera. Exposure time was set at 40–57 ms and the images were taken every 5 s (0.8–1.14% duty cycle) and saved as a TIFF stack file. Recordings were taken for at least 10 minutes of stable baseline before the CNO application (0.5 μM, for 5–6 minutes) followed by 60 minutes washout. At the end of each recording, cells were tested to 6 mM K^+^, raising the concentration from 2.5 to 6 mM. That increase is expected to produce a ~5 mV membrane depolarization and to result to a clear GCaMP florescent increase that was used to compare to the response to CNO.

### Photostimulation and Photoinhibition

Photoinhibition was performed using a yellow-orange light laser (593 nm; LaserGlow) controlled by Spike 8.01 software and patterns consisted of 10 stimuli of 60 seconds delivered at 8–12 mV with a 30 s interval between every stimulus (Machado et al., 2018). Photostimulation was performed using a blue light laser (470 nm; LaserGlow) and the patterns of stimulation (5–20Hz and the time of each stimulation) were controlled by Spike2 8.01 software.

### Thermal imaging

Thermal imaging was performed in *Ptger3*^Cre^ mice during the light phase using an infrared camera (FLIR E4). Before the recordings, the back, neck and head of the mouse was shaved under low dose of ketamine/xylazine. Following at least 2 days recovering from anesthesia and 24h for habituation, mice received injections of CNO or saline, or optogenetic stimulation was performed. Thermal images from pictures and videos recorded the time course changes in BAT temperature (TBAT) and tail temperature (Ttail). Both videos and images were used to determine TBAT (using a mean of the temperature between the right and left scapula) and Ttail (using tree different points from the body to the most distal region of the tail) FLIR tools software was used for all analysis.

### Perfusion and brain sectioning

After performing the experimental protocols mice were deeply anesthetized with chloral hydrate (1.5% BW i.p. 7% solution) and transcardially perfused with 30ml phosphate buffer saline and then 30ml 10% pH neutral formalin (Fischer). Brains were extracted and post fixed overnight in 10% formalin and then stored in 20% sucrose until sectioned using a freezing microtome (30 or 40 μm coronal sections into 3 series). Following sectioning tissue was stored at 4°C in PBS containing the preservative sodium azide until processed for histology.

### Injection sites

In order to confirm the injection sites, one series of the tissue was mounted and cover slipped with hard-set mounting media (Vectashield) for native fluoresce (GFP or mCherry). Only animals with the histological confirmation of the region targeted were used for analyses.

### In situ hybridization

POA sections were used for *Ptger3 (EP3R)* mRNA labelling (RNAscope Probe- Mm-*Ptger3*; catalog #504481, Advanced Cell Diagnostics) or *Adcyap1* (PACAP) mRNA labelling (RNAscope Probe-Mm-Adcyap1; catalog #405911-C2, Advanced Cell Diagnostics, CA). We used a protocol previously established by our group (Machado et al., 2020). At the end of this protocol, brain sections of preoptic region were incubated in TSA plus Fluorescein or Cy5 fluorophores (Catalog # NEL754001KT, Lot#24833936, Perkin Elmer, MA) in 1:1000 concentration for 30 min to visualize (Channel 1 at 488nm) *Ptger3* and *Adycyap1* (PACAP) (Channel 2 at 640nm) mRNA. Slides were dried and cover-slipped with Vectashield mounting medium (Catalog # H-1400, Vector laboratories, CA) before imaging the sections.

### QUANTIFICATION AND STATISTICAL ANALYSIS

#### Quantitative analyses of histology

For all experiments counting number of colocalizations in the MnPO, we counted neuron bodies located at 2 levels of coronal sections (30 μm or 40 μm) from the MnPO. EP3R+ cells in the MnPO region are spread in Y inverted shape around the third ventricle (Fig. 2e–f). For quantification of EP3R and PACAP in this region, we used a rostral coronal section from the beginning of the third ventricle over and around the dorsal cap of the organum vasculosum of the lamina terminalis (OVLT), and a second coronal section for cell bodies ventral to and around the middle of the anterior commissure (ac), the ventral part extended to the upper border of the third ventricle. An Abercrombie correction was applied to all cell counts^29^.

#### Data analysis of calcium imaging data

For the analysis of the calcium imaging signals we used: Fiji (https://imagej.net/software/fiji/)(Schindelin et al., 2012); Suite2P (https://www.suite2p.org/) and MATLAB (MathWorks, Natick, MA) software. TIFF stack files were initially viewed using Fiji software and files with change in focus were discarded. Image registration by phase correlation, automatic cell detection and neuropil fluorescent signal calculation were done using Suite2P software. Regions of interest (ROIs) that included two or more cells or cases in which multiple ROIs sampled the same cell were manually deleted. The ROI fluorescent signals were further processed in MATLAB for the corrections of the fluorescence background. Background correction was applied to each ROI by subtracting 70% of the cell neuropil signal. ROIs fluorescent data were then normalized by Z-score with baseline selection (F-(mean[F_baseline_])/SD[F_baseline_]) with F_baseline_ as fluorescent signal during the 10 min recordings before CNO application. Z-score changes during KCl application (*n* = 261 cells) were ordered from lowest to highest values and only neurons that had a change in Z-score greater than the first quartile were used for analysis^30^.

#### Statistical analysis

Statistical analyses were performed using GraphPad Prism v7 (GraphPad Software). Following a test for normality (D’Agostino and Pearson omnibus test), significant differences were determined using either repeated measures one-way ANOVA or two-way ANOVA both followed by Bonferroni’s correction or for statistical tests between two groups a two-sided t-test was used. If the data did not pass the normality test, the Kruskal-Wallis test, followed by Dunn’s post hoc test was used. Data are presented as mean ± SEM. The statistical test used, statistical significance and number of animal subjects per group are reported in the results and figure legends, data were considered to be statistically significant when p<0.05.

## Supplementary Material

1

## Data Availability

The data that support the findings in this study are available from the corresponding author upon request.
