# Supplementary material for: Prolonged activation of EP3 receptor-expressing preoptic neurons underlies torpor responses
Source: Res Sq. 2023 May 2:rs.3.rs-2861253. Preprint. [Version 1] doi: 10.21203/rs.3.rs-2861253/v1 (PMC10187418; doi:10.21203/rs.3.rs-2861253/v1)
Supplement: 1 [file NIHPPRS2861253V1-supplement-1.pdf]

Fig. S 1

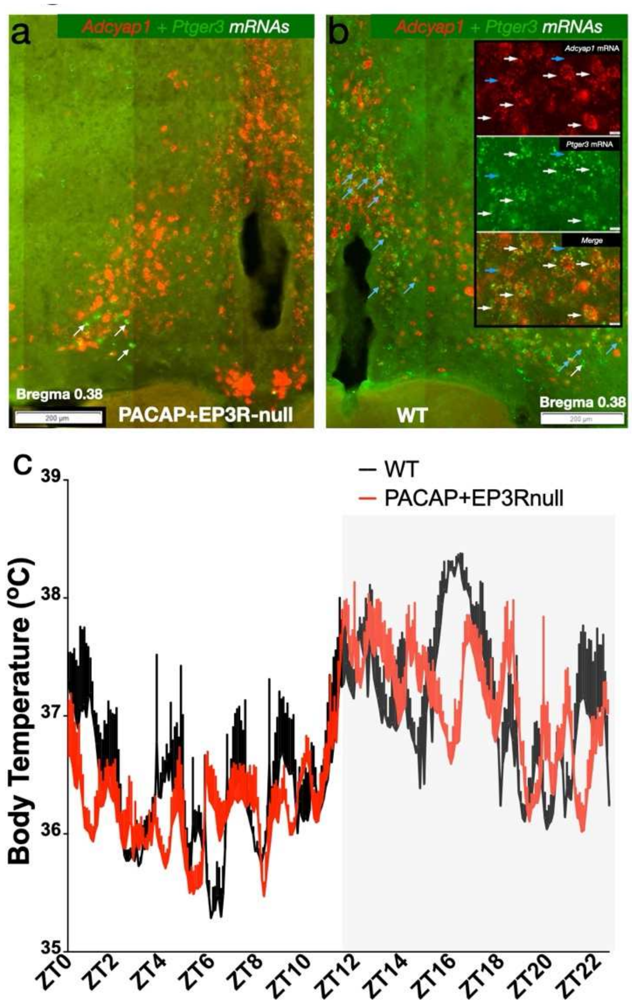

Fig S 1. Deletion of EP3R from PACAP-expressing neurons does not change baseline body temperature.

We crossed *Ptger3*$^{flox/flox}$ mice with *Adcyap1*^{Cre} mice to delete EP3R from PACAP+ neurons (PACAP+EP3R-null mice). RNA-scope in situ hybridization showed successful deletion of EP3R (*Ptger3*, green) from PACAP-expressing (*Adcyap1*, red) MnPO neurons (n=2), and showed a remaining small subset of MnPO$^{EP3R+/PACAP-}$ neurons (white arrows) in PACAP+EP3R-null mice (a). In situ hybridization in a WT mouse showed a high degree of colocalization of *Ptger3* and *Adcyap1* mRNAs (blue arrows) in the MnPO (a, on the right panel)). The WT (n=3) and PACAP+EP3R-null mice (n=5) showed similar Tb baseline at 22 ºC ambient temperature during the light and dark phase (mean Tb 36.7 ºC ± 0.02 in WT mice vs. 36.6 ºC ± 0.02 in PACAP+EP3R-null mice. $F_{(288, 1680)}$ = 1.169, One-way ANOVA, followed by Bonferroni's post hoc test, $p>0.99$) (b).

Fig. S 2

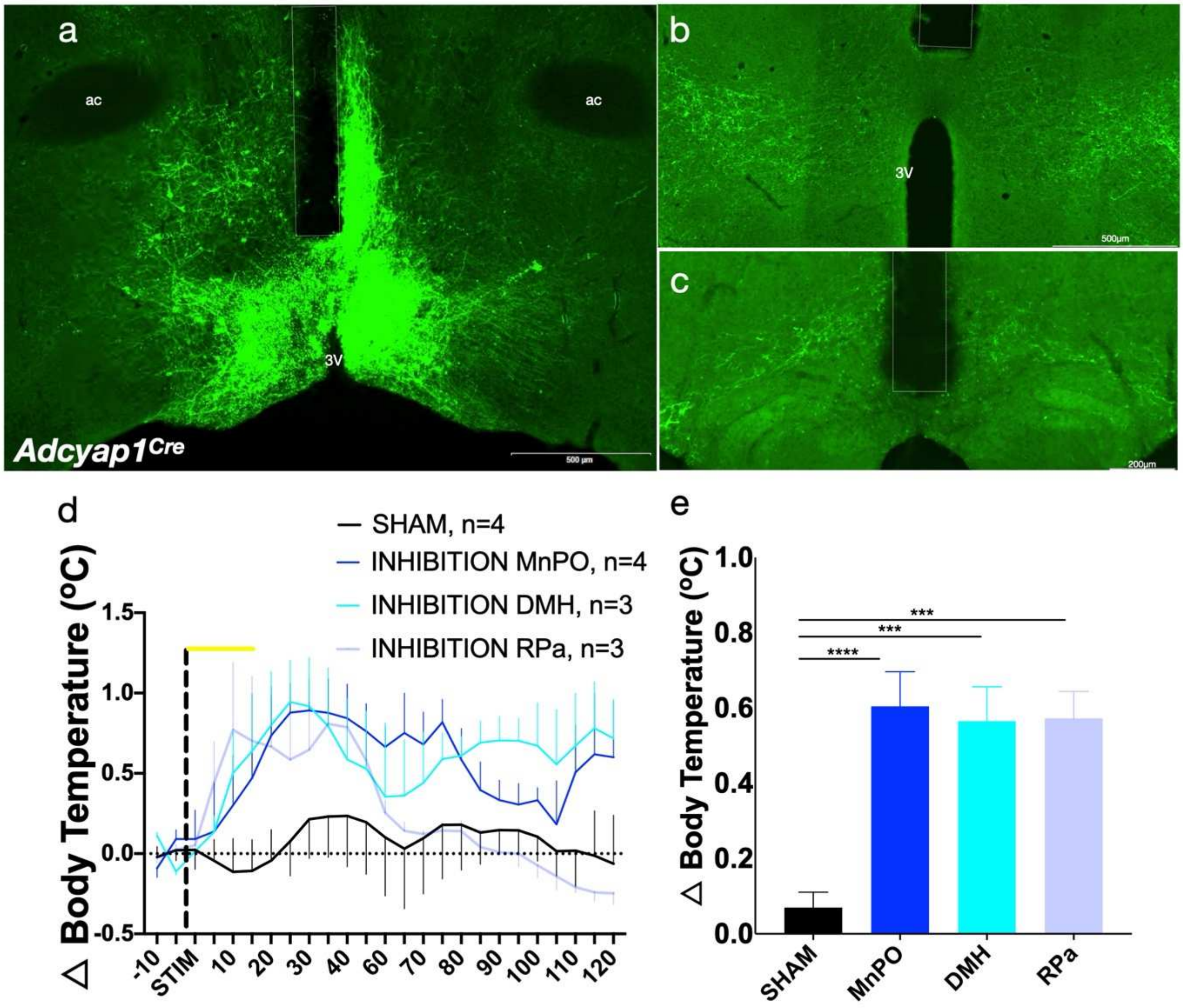

Fig. S 2. MnPO^{PACAP+} neurons project to the dorsomedial hypothalamus and raphe pallidus to modulate body temperature.

Representative fluorescence images of ArchT-GFP+ expression and optical fiber placement over MnPO neurons of a *Adcyap1^{Cre}* mouse (a) and the terminals of MnPO^{PACAP} neurons in the DMH (b) and RPa (c). Photoinhibiting MnPO^{PACAP} cell bodies (n=4) or their synaptic terminals in the DMH (n=3) or RPa (n=3) for 15 min (10 repetitions of laser stimulation for 60 s, 8-12 mV, followed by 30-s laser “off” intervals) caused a hyperthermic effect that lasted for over two hours when inhibiting MnPO^{PACAP} cell bodies or their synaptic terminals in the DMH, but only for about 1 h when the stimulation was delivered in the synaptic terminals in the RPa (d). During the first hour from the onset of photoinhibition we found a comparable elevation of Tb in all experimental groups compared to sham stimulation (0.60 ± 0.09°C SEM MnPO STIM vs 0.56 ± 0.04°C SEM DMH STIM vs 0.57 ± 0.07°C SEM RPa STIM vs 0.06 ± 0.04 °C SEM SHAM STIM, $F_{(3,40)}$=11.3, One-way ANOVA, followed by Bonferroni's post hoc test, $p<0.0001$) (e).

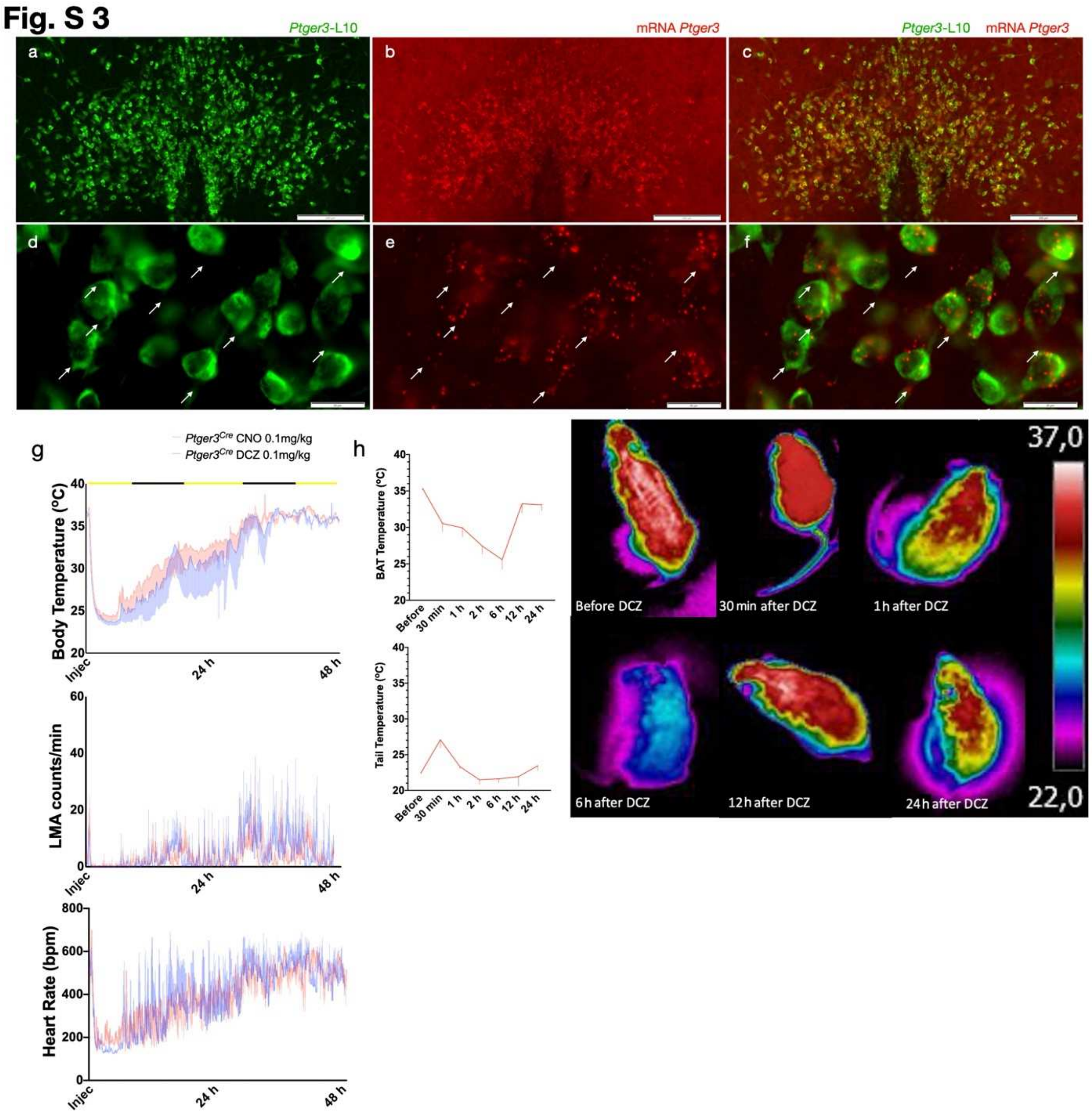

Fig S 3. A chemogenetic ligand of short half-life promotes activation of EP3R-expressing MnPO neurons and induces a prolonged deep hypothermia.

EP3R-expressing MnPO neurons of a *Ptger3*^{Cre}::L10-GFP mouse (n=2). *Ptger3*-L10 neurons are green (a), *Ptger3* mRNA is red (b) and the colocalization of *Ptger3*- L10 and *Ptger3* mRNA is seen in the merged image (c). At higher magnification, most (white arrows) but not all green *Ptger3*-L10 neurons show red *Ptger3* mRNA, which may reflect lower sensitivity of in situ hybridization, or that some *Ptger3*-L10 neurons expressed EP3R during development but no longer do so (d-f). Mice treated with CNO (0.1mg/kg) (pink) or DCZ (light blue) also showed similar hypothermic responses (mean Tb was 32.4 ± 0.16 °C after CNO vs. 31.4 ± 0.18 °C after DCZ during the first 48h after drug

treatment, t-test, $p<0.0001$, n=4 and 3 respectively). During the 2 days (48 hrs) following the injection, both CNO and DCZ caused a similar decrease of the mean of the locomotor activity (3.3 ± 0.15 counts per minute after CNO vs. 4.29 ± 0.2 counts per minute after DCZ, t-test, p=0.0003, n=4 and 3 respectively) and bradycardia (403 ± 4.9 bpm *Ptger3*^{Cre} CNO vs. 378 ± 6.0 bpm *Ptger3*^{Cre} DCZ, t-test, $p<0.0001$, n=4 and 3 respectively) (g). Prior to DCZ, the surface temperature over the BAT just behind the head and in the paraspinal region was around 37 ^oC (white) and the tail was close to ambient temperature (22 ^oC, magenta to black). By 30-60 min after DCZ, the tail was warmer (blue, about 27 ^oC) and the body was already cooling (about 32 ^oC in the interscapular region). By 6 h, the skin temperature over the back was about 27 ^oC and the tail around ambient temperature, followed by gradual but incomplete recovery from 12-24 hr (n=3; h).

Fig. S 4

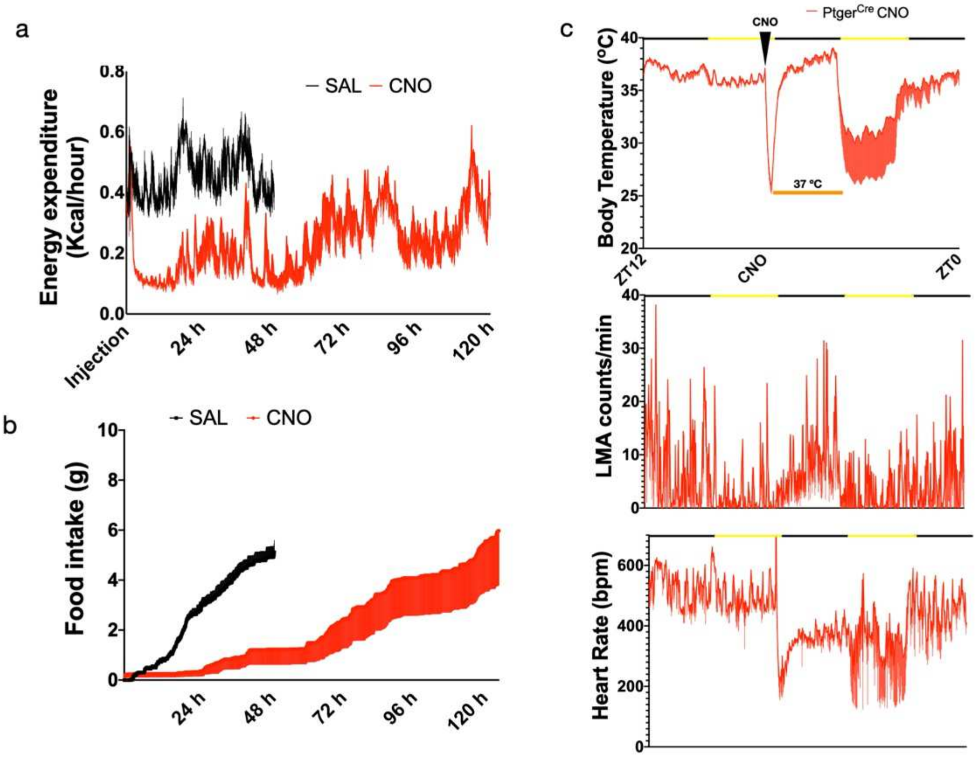

Fig S 4. Temporary reversal of hypothermia caused by chemogenetic activation of EP3R-expressing MnPO neurons by warm environmental conditions.

After CNO injection, some mice required up to 72 h to return to baseline levels of food consumption (a) and over 120 h to return to normal levels of energy expenditure (n=4) (b). Hypothermia caused by chemogenetic activation of MnPO^{EP3R} neurons (n=3) was temporarily reversed by placing the mice in a 37°C environment for 12 hours; this also increased LMA, but the HR remained at a reduced level (c), suggesting a continued reduced metabolic rate. When the mice were returned to 22°C ambient temperature, Tb and LMA immediately fell back to levels like those before warming.

Fig. S 5

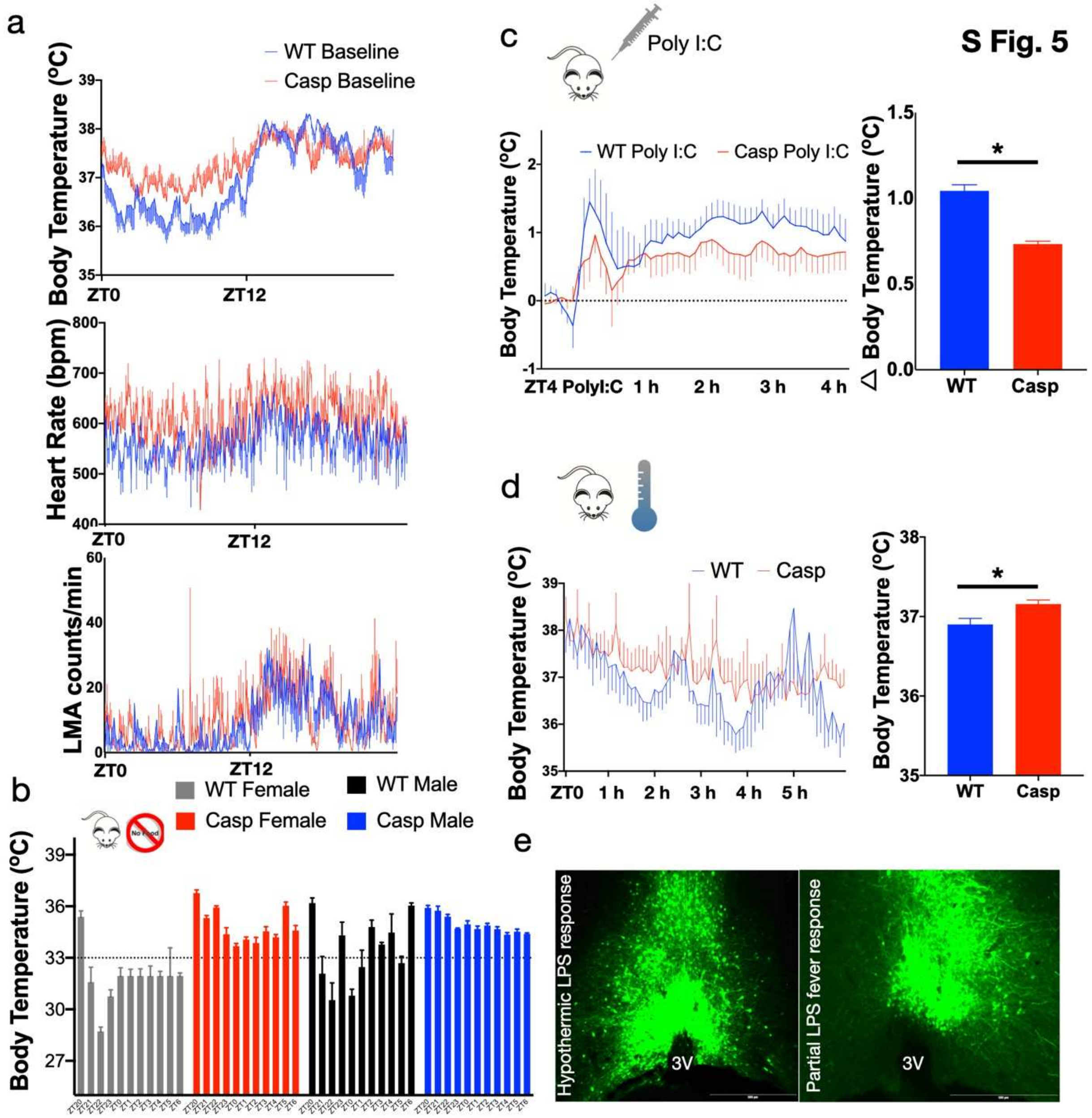

Fig S 5. MnPO^{EP3R} neurons participate in diurnal regulation of Tb, in the fever induced by PolyI:C, and in cold-induced thermoregulatory adaptation.

The baseline Tb of *Ptger3*^{Cre} and WT (control) mice injected with AAV-DIO-casp in the MnPO show that deletion of MnPO^{EP3R} neurons produced an inappropriately high basal Tb and HR during the light period but not the dark period and caused minimal increases in the LMA of mice across the entire day (a). In response to food deprivation (FD) beginning at lights out (ZT12), intact WT male and female (n=6 each) mice showed torpor bouts, with Tb dropping below 33 °C. Mice with deletion of MnPO^{EP3R} neurons showed a gradual fall in Tb but from ZT21 onward, but failed to produce torpor

responses (b). After injection of poly I:C, which triggers an innate immune response and fever, we found that $MnPO^{EP3R}$-ablated mice show an attenuated fever response compared to WT controls (1.0 ± 0.04 °C SEM WT poly I:C vs 0.7 ± 0.02 °C SEM Casp, n=5 each group, unpaired t-test $p<0.0001$) (c). In the absence of $MnPO^{EP3R}$ neurons, mice show a slightly smaller fall in Tb in response to a cold (4°C) environment (36.9 ± 0.07°C SEM WT 4°C vs 37.2 ± 0.05°C SEM Casp, n=5 each group, unpaired t-test $p=0.005$) (d). To monitor placement of injections of AAV-DIO-caspase 3, we co-injected AAV-GFP. In three animals with injections successfully targeting the $MnPO^{EP3R}$ neurons (e, left), animals showed a hypothermic response to LPS, like that seen in animals with complete EP3R deletion in the preoptic area. In two animals with more dorsally placed partial injections (e, right), animals had a reduction but not elimination of hyperthermia after LPS.

Fig S 6

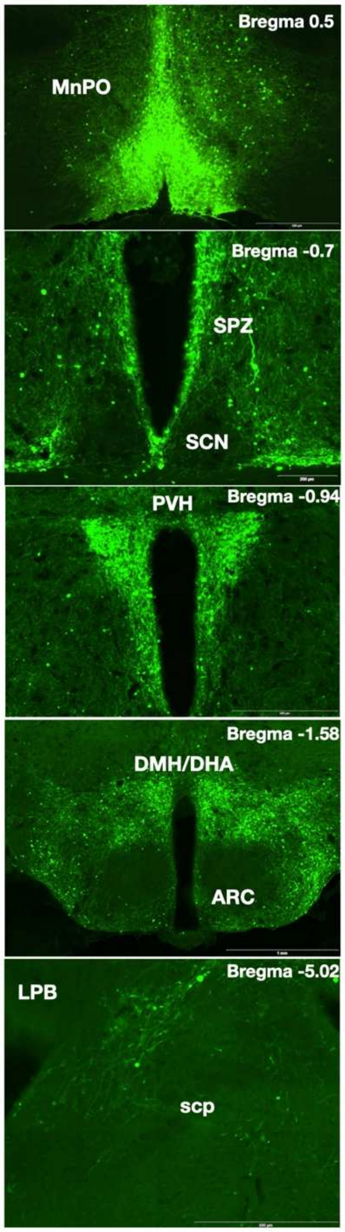

Brain Regions	
Dorsolateral preoptic nucleus	++++
Ventrolateral preoptic nucleus (VLPO)	++++
Bed nucleus of the stria terminalis (BNST)	+++
Subfornical organ (SFO)	+++
Suprachiasmatic nucleus (SCN)	++
Subparaventricular zone of the hypothalamus (SPZ)	++
Supraoptic nucleus (SON)	++
Amygdala	++
Paraventricular hypothalamic nucleus (parvocellular)	+++
Periventricular hypothalamic nucleus	++++
Paraventricular hypothalamic nucleus (magnocellular)	++++
Paraventricular hypothalamic nucleus (posterior)	++++
Paraventricular nucleus of the thalamus (PVH)	+++
Lateroanterior hypothalamic nucleus (LA)	++
Reuniens thalamic nucleus	++++
Xiphoid thalamic nucleus	++++
Lateral hypothalamus (LH)	+++
Perifornical area (PeF)	+++
Median eminence (ME)	+
Dorsomedial hypothalamus (dorsal) (DMD)	+++
Dorsomedial hypothalamus (ventral) (DMV)	++
Dorsomedial hypothalamus (compact) (DMC)	+
Arcuate nucleus (Arc)	++
Tuberomammillary nucleus	++
Premammillary nucleus	+
Dorsal and dorsolateral periaqueductal gray	+
Dorsal raphe and median raphe (DR/MnR)	+++
Pedunculopontine tegmental nucleus (PTg)	+
Ventrolateral periaqueductal gray (VLPAG)	++
Barrington's nucleus/laterodorsal tegmental nucleus (Bar/LDTg)	++
Lateral parabrachial nucleus region (LPB)	++
Locus coeruleus (LC)	++
Raphe pallidus (RPa)	++
Parapyrimidal region (PPy)	+
Raphe obscurus (Rob)	+
Commissural nucleus of the solitary tract/central canal	+

Sparse innervation +
Sparse-moderate innervation ++
Moderate-dense innervation +++
Dense innervation ++++

Fig S 6. A qualitative summary of the density of terminals for $MnPO^{EP3R}$ neurons based on cell-specific anterograde tracing.

Cre-dependent ChR2-YFP injection into the MnPO of *Ptger3*^{Cre} mice permitted a selective map of the projections from EP3R-expressing MnPO neurons, n=4. + = sparse terminal labeling; ++ = sparse to moderate labeling, +++ = moderate to dense labeling, ++++ = dense labeling.
